# Notes and News

**Published:** 1903-06-06

**Authors:** 


### Notes and News.

On Tuesday next the Princess of Wales will open the New Home for the Nurses at the British Lying-in Hospital.

The Duchess of Albany and Princess Alice of Albany will attend the concert in aid of the League of Mercy at Grosvenor House on Friday.

The Metropolitan Asylums Board have established their first Ophthalmic Hospital and School at Swanley.

Commemoration Day of the Livingstone College for Medical Missionaries will be celebrated on June 10th at the College Leyton.

At a meeting of the British Gynæcological Society, to be held at 20 Hanover Square, on Thursday, the 11th inst., at 8 p.m., Dr. C. Routh will read a paper on Some Directions and Avenues through which probably a more Successful Treatment of Cancer may Result, and Dr. Mansell Moullin will read a paper on the Treatment of Hæmatocolpos and Hæmatometra.

Dr. Florence Sabin, Assistant in Anatomy at the Johns Hopkins University School at Baltimore, has gained the £200 prize offered for the best piece of scientific research work in America during the past two years. Dr. Florence Sabin is to be congratulated on her success, as whilst women frequently shine in the field of hard work, they have so far seldom distinguished themselves for originality

Many people are seeking a comfortable Home for consumptive relatives where they can secure proper care and treatment without institutional surroundings. These requirements are difficult to satisfy, but we are glad to call the attention of our readers to the Home over which Mrs. White, a hospital matron of long standing, presides. The address is Bronwylfa, Penmaenmawr, in Wales. The air at Penmaenmawr is excellent, and the train service is good.

The Nottingham Hospital is the recipient of a legacy of £1,000 from the late Arthur Shrewsbury, the well-known cricketer.

The number of small-pox cases under treatment in the Metropolitan Asylums Boards remains about the same, and numbered about sixty last week.

The Harveian Festival in Edinburgh this year will be held on June 5th. Professor John Chiene, the President, will deliver the oration "On the Early Days of the Edinburgh Royal Infirmary."

The forty-third annual dinner of King's College, London, will be held at the Holborn Restaurant, on Monday, June 22nd, with the Right Hon. and Right Rev. the Lord Bishop of Exeter in the chair.

A Board of Guardians in Norfolk endeavoured to secure the re-vaccination of tramps seeking admission to the workhouse. They were unable to compel the tramps to submit, and the medical officer was unable to persuade any of them to consent, so the effort was abandoned. Possibly an order enforcing tramps to be re-vaccinated when it was deemed necessary would serve the twofold purpose of reducing dependence on Poor Law relief and checking the spread of smallpox.

A great deal of interest is being excited in Hospital Sunday this year owing to the intended visit of the King and Queen to St. Paul's Cathedral. Doubtless thousands of people who cannot obtain admission to the Cathedral will assemble on the Royal route. A correspondent writes and suggests that under proper organisation the occasion might legitimately be utilised to reap a harvest for the hospitals through a collection from the spectators, but even under the present circumstances we cannot advocate the collecting box.

It is reported that an officer in the Punjaub has put in a claim for a pension owing to the injury he has suffered from neurosis of his right hand cause by excessive clerical work. The question of compensation for injuries arising from overwork in any employment is a difficult one. No doubt that permanent harm more frequently results from overwork than from accident, but a claim for compensation has hardly ever been raised under this plea as the difficulties to establish the justice of such claims are too great.

The subject of consumption provokes so much interest amongst all classes of the community that it is now frequently the basis of articles in the popular magazines. The *Pall Mall Magazine* for June has a very interesting article signed "By One Who Has Been Cured," giving an illustrated and detailed account of the writer's experience and treatment in a Scotch sanatorium which he prefers not to name, but which nevertheless is not difficult to locate. The curiosity of readers will be thoroughly satisfied for the writer's actual filled-in diet sheet, temperature chart, and record of exercise are published.

